# Sex-specific spatial and temporal gene expressions of Pheromone biosynthesis activating neuropeptide (PBAN) and binding proteins (PBP/OBP) in *Spoladea recurvalis*

**DOI:** 10.1038/s41598-019-39822-x

**Published:** 2019-03-05

**Authors:** Rajendran Senthilkumar, Ramasamy Srinivasan

**Affiliations:** World Vegetable Center, Shanhua, Tainan 74151 Taiwan

## Abstract

*Spoladea recurvalis* is one of the most destructive insect pests of amaranth, a leafy vegetable in both Asia and Africa. The present study characterized the pheromone biosynthesis-activating neuropeptide (DH-PBAN) and pheromone/odorant binding proteins in *S. recurvalis*. The open reading frame of 600 base pairs encodes a 200-amino acid protein possessing five neuropeptide motifs (DH, PBAN, α-, β-, and γ- subesophageal ganglion neuropeptides) and shares a characteristic conserved C-terminal pentapeptide fragment FXPRL. The full-length genome of Spre-DH-PBAN was 4,295 bp in length and comprised of six exons interspersed by five introns. Sequence homology and phylogenetic analysis of Spre-DH-PBAN have high similarity to its homologs in Crambidae of Lepidopteran order. We quantitatively measured the relative expression level (qRT_PCR) of Spre-DH-PBAN gene, the binding proteins such as odorant binding proteins (OBPs) and pheromone binding protein (PBPs) at different developmental stages. The results confirmed their role in recognition and chemoreception of sex pheromone components, and they were distinct, tissue- and sex-specific. This is the first report on the molecular analysis of PBAN gene and binding proteins, which can improve the understanding of molecular mechanisms of growth, development, and reproductive behavior of *S. recurvalis*, and may become effective targets for controlling this insect.

## Introduction

Physiological processes such as diapause, mating, eclosion, reproduction and metamorphosis are determined by neurohormones, known as insect neuropeptides^1^. Female insects produce pheromones to entice sexual communication between male and female moths over long distances, which are recognized by a distinct neural arrangement in male moths, and eventually processed for mating^2^. Mostly, species-specific sex pheromones are produced in female pheromone glands (PG) located on the terminal abdominal segments in most of the moth species, which are released to attract conspecific males for mating^2,3^. The Pyrokinin (PK)/pheromone biosynthesis-activating neuropeptide (PBAN) helps in regulating the sex pheromone production in insects, especially lepidopteran species in particular^4,5^. Based on the studies of molecular characterization, DH-PBAN is a multigene family, encoded by five putative neuropeptides – PBAN, DH, *α*-, *β*- and *γ*- subesophageal ganglion neuropeptides (SGNP). These are all well conserved C-terminal FXPRL amide motif, and are released through post-translational processing^5,6^. Although physiological functions such as melanization in moth^7^, regulation of pheromone biosynthesis^8^, and stimulation of embryonic diapause in insects like silkworm moth^9^ were well documented, their functions were not aptly understood. The differing physiological functions, reported previously, were controlled by the wide distribution and expression of FXPRL family peptide encoded by PBAN^10–12^. PBAN serves to regulate the production and release of sex pheromones in female moths^13^. In *Helicoverpa zea*, a 33 amino acid C-terminal amidated peptide of PBAN was first identified^8^, whereas the determination of its amino acid sequence was done by direct isolation and purification from the *Bombyx mori*’s head^14^. Later, PBAN encoding genes were identified in *Aedes aegypti*^15^, *Ostrinia nubilalis*^16^, *Chlumentia transversa*^17^, *Maruca vitrata*^18^, *Manduca sexta*^19^, and *B. mori*^20^. As a result, DH-PBAN proteins have been reported for more than 30 insect species spanning four orders including Lepidoptera and Hymenoptera^21^.

The pheromone binding proteins (PBPs) penetrate into an aqueous layer of sensillum lymph, transported to olfactory receptor neurons, where signal transduction is initiated^22–24^ and thus mediating perceptions of pheromones in the moths. In fact, PBPs are the sub-class of insect odorant binding proteins (OBPs)^25^. PBPs were initially discovered in Lepidoptera and later characterized in a series of insect orders including Diptera, Hymenoptera, Hemiptera and Coleoptera^26,27^. Studies on PBPs indicated that they concentrate odorants in the sensillum lymph, act as solubilizers and carriers of the hydrophobic pheromones, involves in odorant molecule deactivation, serve as co-factors in the activation of pheromone receptors, and protect the pheromone from enzymatic degradation^28^. In fact, absence of PBPs affects the sensitivity of the neuron to its odor ligand as reported previously^29^. OBP sequences possess conserved cysteine residues, and are classified based on their number and location such as “classic”, “plus-C”, “minus-C” and “duplex”^30^. However, most pattern of the cysteine residues in the amino acid sequences pose a signature for moth OBPs, and are differing^31^. Among the many OBP classes, the six-cysteine residues are the most pertinent in the process of PBPs folding^26,32^. Therefore, it is evident that the olfactory recognition system serves an important role in many processes like orientation, oviposition, mating, feeding, and in searching for hosts, rather than its primary role of ensuring reproduction and insect survival^33^.

The beet webworm, *Spoladea recurvalis* Fab. (Lepidoptera: Crambidae) mainly inhabits the tropical and sub-tropical areas of the world, where it causes severe damage to crops including bean, amaranth, cucurbits, beetroot, sweet potato, and eggplant^34^. It was shown that *S. recurvalis* can migrate long distances causing serious crop destruction in new locations^35^. Studies on host-plant interactions and bio-ecology of *S*. *recurvalis* showed serious consequences in amaranth^36^. Contrary to the use of chemical pesticides that pose serious risks to environment and human health, use of pheromones to interrupt the mating behavior is deemed to be the most appropriate component in integrated pest management (IPM) packages.

In light of this, we characterized the pheromone biosynthesis activating neuropeptide and binding proteins of *S. recurvalis* to perform the tissue-specific DNA expression analyzes in both male and female moths with the aim of using the proteins as primary molecular targets for designing and developing new pest management strategies in future studies.

## Results

### Structure of *Spoladea recurvalis* PBAN (Spre-DH-PBAN) Gene

**Figure 1:**
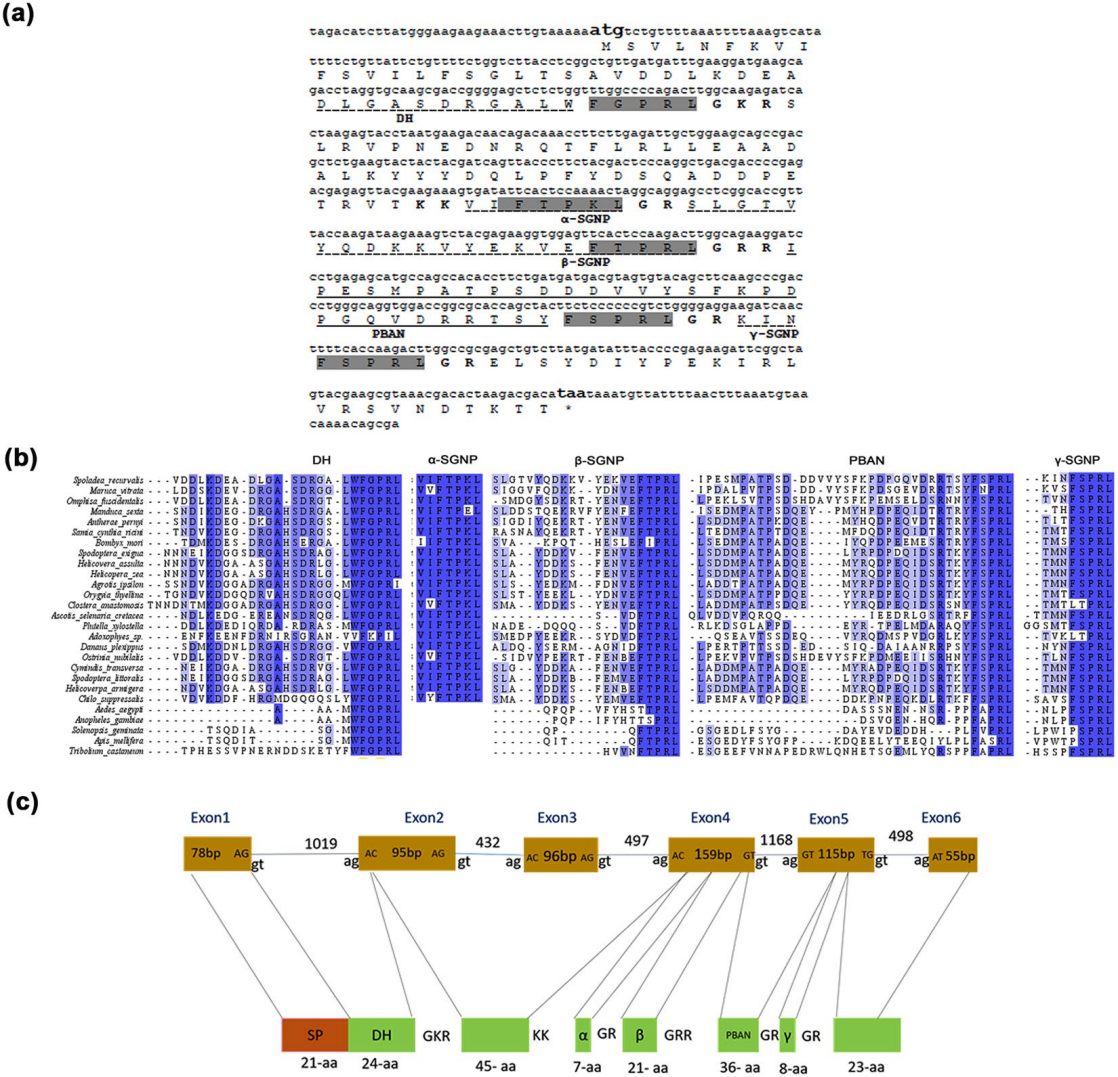
Molecular characterization of gene encoding pheromone biosynthesis activating neuropeptide (DH-PBAN) gene in *Spoladea recurvalis*. (**a**) The putative start codon (ATG) and stop codon (TAA) are indicated in bold font. Amino acids of five putative FXPRL-NH_2_ peptides are underlined: diapause hormone (DH), α,β,γ- suboesphageal ganglion neuropeptide (SGNP) and PBAN conserved C-terminal pentapeptide sequences are highlighted in bold letters with grey background. (**b**) Multiple sequence alignment of consensus sequences of diapause hormone-pheromone biosynthesis activating neuropeptide (DH-PBAN) precursor from *Spoladea recurvalis* and other lepidopteran species. Percentage of conserved amino acids are highlighted in blue. (**c**) DH-PBAN Exons are represented at the top solid boxes with boundary sequences. Horizontal lines between exons are represents intronic regions with boundary sequences. FXPRL-NH_2_ neuropeptides along with signal peptide are represented at the bottom solid box. GKR;KK;GR;GRR denotes endoproteolytic cleavage site; SP-signal peptide; DH-diapause hormone; SGNP neuropeptides.

**Figure 2:**
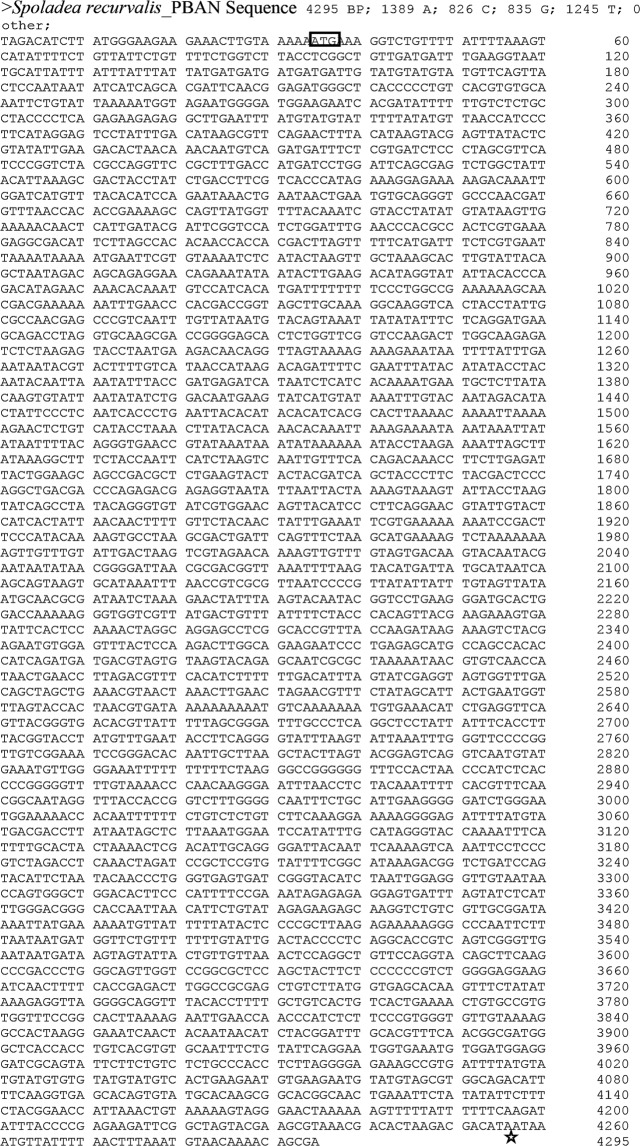
Exon-intron organization and complete nucleotide sequence *of Spoladea recurvalis* DH-PBAN gene. DNA sequences of 4.2 kb segment of DH-PBAN. Nucleotide sequences encoded by the exon are highlighted in blue highlights. Start codon indicates in box and stop codon indicated in .

The full-length transcript of 672 bp, which comprised of the 34 bp 5′ Untranslated region (5′UTR), 600 bp Open reading frame (ORF), and 38 bp 3′ Untranslated region (3′UTR) was identified from the *S. recurvalis* transcriptome library. The 600 bp nucleotides in the ORF encode 200 amino acids, possessing five putative neuropeptides namely, β-SGNP, PBAN, DH, α-SGNP, and γ-SGNP from 5′ end to 3′ end (Fig. 1a). The polypeptide that comprises 21 amino acids was predicted to be a putative signal peptide with cleavage site between first M1 and V22 amino acids. Figure 1b indicated that the Spre-DH-PBAN showed high similarities of 80% to its orthologs of Crambidae from Lepidoptera, and has the lowest similarities of 32% to the DH-PBAN proteins of the family Culicidae from Diptera. The amino acid sequences of individual neuropeptides such as γ-SGNP, α-SGNP, DH, β-SGNP, PBAN and the full length DH-PBAN showed 71–100%, 85–100%, 52–100%, 56–100%, 40–78%, and 30–80% identities, respectively, with all the compared insect species (Table 1). The highest percentage identity was shown for α-SGNP and γ-SGNP, followed by DH and β-SGNP. Schematic comparison of the genomic DNA and the translated protein structure of *S. recurvalis* DH-PBAN are shown in Fig. 1c. The putative signal peptide and the N-terminal portion DH peptide were translated from exon 1 and 2 (95 bp) and encoded 45 amino acids. Markedly, neuropeptides β-SGNP, partial PBAN, and α-SGNP, which were translated from the exon 4 (153 bp) encoded 51 amino acids. The γ-SGNP and PBAN were translated from exon 5, encoding 37 amino acids that cover the C-terminal ends (Fig. 1c). The intron-exon of Spre-DH-PBAN gene was approximately 4,295 bp in length containing 6 exons interspersed by 5 introns. The grouping of the corresponding genomic architecture and cDNA were summarized in Fig. 2. Untranslated regions (UTR) of 33 nucleotides start from upstream of 5′, the start site ATG of exon 1 (81 bp) (Fig. 2). All progressions of the exon/intron borders were in agreement with the consent GT/AG series to both the acceptor and donor sites of the gene splicing. The intron sizes range from 432–1,168 nucleotide structural components, except for the intron between exon 4 and exon 5 with the largest proportion of 1,168 nucleotides in length, spanning PBAN neuropeptide (Fig. 2).

**Table 1:** Percentage of amino acid sequences homology of five neuropeptide and full-length DH-PBAN homolog to *Spoladea recurvalis* inferred from species of four orders (Lepidoptera, Coleoptera, Diptera and Hymenoptera).

Species	Neuropeptide (percentage % homology)						Accession Number
	DH	α- SGNP	β-SGNP	PBAN	γ-SGNP	FL DH-PBAN	
*Spoladea recurvalis*	—	—	—	—	—	—	
*Maruca vitrata*	82.3	85	71	77.7	75	80.2	AFX71575
*Omphisa fuscidentalis*	91.6	100	75	58	85	74.8	AFP87384
*Manduca sexta*	79.1	85	68	62	100	63.4	AAO18192
*Antheraea pernyi*	75	100	66	59	85	63.64	AAR17699
*Samia cynthia ricini*	75	100	75	55.5	—	62.7	AAP41132
*Bombyx mori*	78.2	85	66	50	100	62.6	AAB24327
*Spodoptera exigua*	51.7	100	75	52.7	100	64.3	AAT64424
*Helicoverpa assulta*	66.6	100	68	50	100	65.7	AAC64293
*Helicoverpa zea*	66.6	100	68	50	—	65.7	AAA20661
*Agrotis ipsilon*	66.6	100	62	50	100	62.5	CAA08774
*Orgyia thyellina*	61.5	100	62	52.7	100	63.6	BAE94185
*Clostera anastomosis*	73.9	85	75	50	—	60.2	ABR04093
*Ascotis selenaria cretacea*	55.5	100	85	40	100	53.8	BAF64458
*Plutella xylostella*	64	100	85	60	100	53.2	AAX99220
*Adoxophyes sp*	—	—	56	44.1	—	53.8	AAK72980
*Danaus plexippus*	64	100	71	47.2	100	52	OWR44299
*Ostrinia nubilalis*	83.3	85	58	59.4	100	70	AOY34029
*Chlumetia transversa*	66.6	100	66	50	100	63	AIY72749
*Spodoptera littoralis*	51.7	100	68	52.7	100	63	AKT95050
*Helicoverpa armigera*	66.6	100	62	50	100	65	AF492474
*Chilo suppressalis*	100	85	75	66.6	71	53	ALM30314
*Aedes aegypti*	87.5	—	100	—	100	32	XP001662212
*Anopheles gambiae*	87.5	—	—	—	100	30	XP307885
*Solenopsis geminata*	100	—	75	—	—	33	ADI88479
*Apis mellifera*	85.7	—	83	—	100	—	A8CL69
*Tribolium castaneum*	100	—	85	—	100	30	EFA11568

### Phylogenetic Relationship of Spre-DH-PBAN

**Figure 3:**
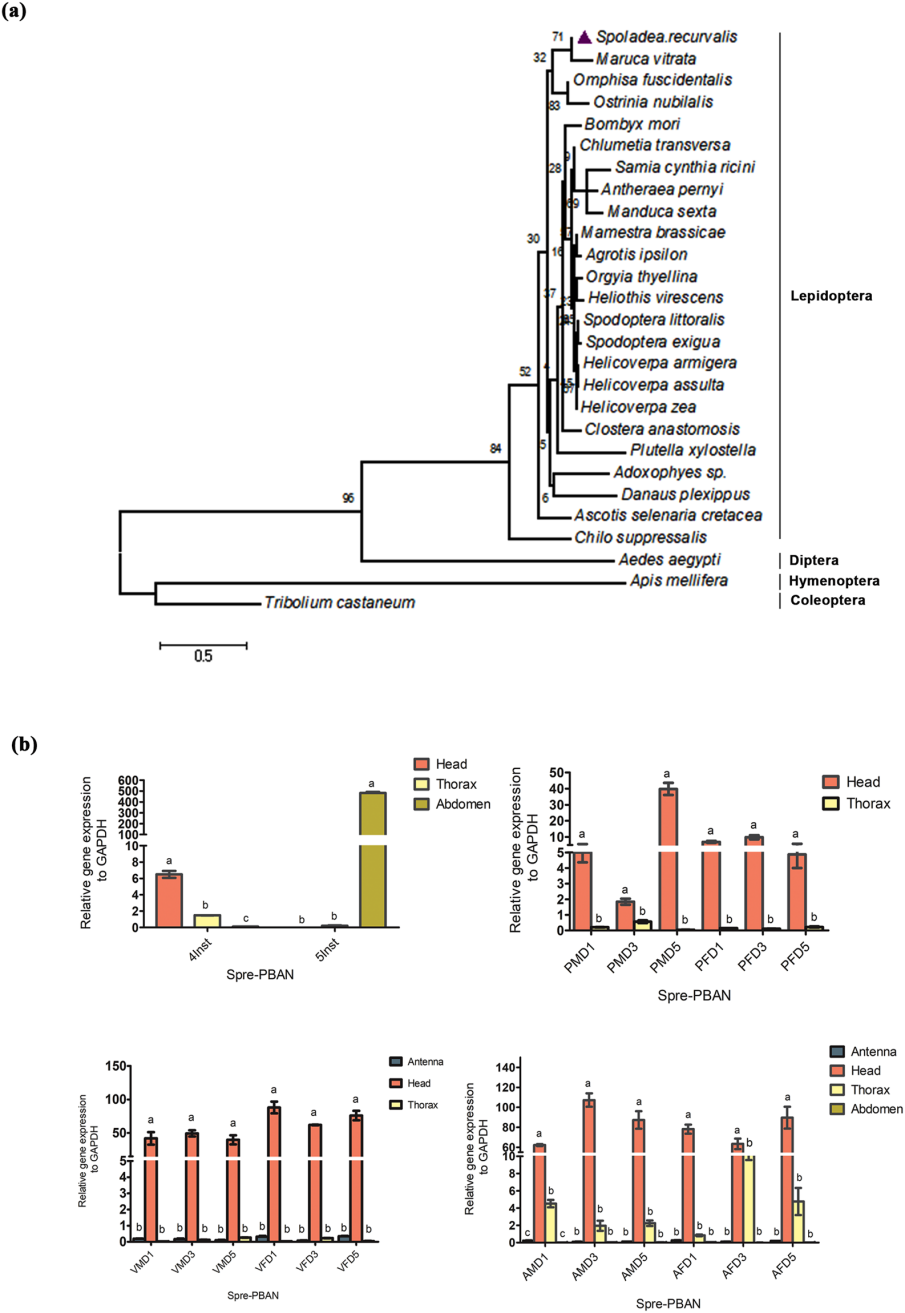
Phylogenetic analysis and Gene expression of PBAN during different developmental stages. (**a**) Maximum likelihood phylogeny tree was constructed using known and putative PBAN amino acid sequences inferred using four insect orders determined by using MEGA 6.0 with default settings model JTT + T inferred from 1000 replicates. (**b**) PBAN gene expression in the development of *S. recurvalis*. Total RNA was prepared at day 1, day 3 and day 5 of each stages Larvae, Pupae, Virgin (male and female), Adult (mated male and female) subjected to qRT-PCR. The results are shown as the Spre-PBAN gene expression level was normalized related to each development stages. GAPDH was used as internal control. Bar represents the ±standard error. Different letters (**a**,**b**,**c**) shows the significant differences (P < 0.05). (Inst-Instar; PMD-Pupae male day; PFD-Pupae female day; VMD-virgin male day; VFD-virgin female day; AMD-Mated adult male day; AFD-Mated adult female day).

The interspecific phylogenetic relationships based on the DH-PBAN ORFs of *S*. *recurvalis* and the 25 other available insect DH-PBAN peptide sequences derived from NCBI GenBank were shown in Fig. 3a. The phylogram based on JTT matrix model was consistent with the taxonomic classification of insects. *Spoladea recurvalis* clustered together with *M*. *vitrata*, *C. supperssalis* and *O. fuscidentalis* (Lepidoptera: Crambidae). The members of the order Lepidoptera form a separate clade from the Diptera (*A. aegypti*), Coleoptera (*Tribolium castaneum*) and Hymenoptera (*Apis mellifera*).

### Gene Expression of DH-PBAN during Developmental Stages

Tissue-specific gene-expression profile of DH-PBAN was done using samples obtained from various life-stages of *S. recurvalis* using qRT-PCR. The abdomen of the 5^th^ instar larva showed higher expression of PBAN compared to the other tissues, for instance head and thorax (Fig. 3b). Then, PBAN expression was found to be high in the head and thorax tissues of male and female pupae. However, only a basal level of expression was observed in the thorax tissues of male and female pupae irrespective of their ages. Furthermore, PBAN gene expression was noticeably higher in the female and male head of unmated adults, from the day 1 to 5, respectively. The PBAN gene expression was also found to be abundant during day 1, and the level of expression was consistently higher from day 3 to 5 of the male and female head tissues in mated adults (Fig. 3b). On contrary, mated adult thorax tissues showed moderate PBAN expression in both male and female of the same age. There was no significant gene expression noticed in abdomen tissues virgin and mated adults of all the ages (Fig. 3b).

### Pheromone/Odorant Binding Proteins

**Figure 4:**
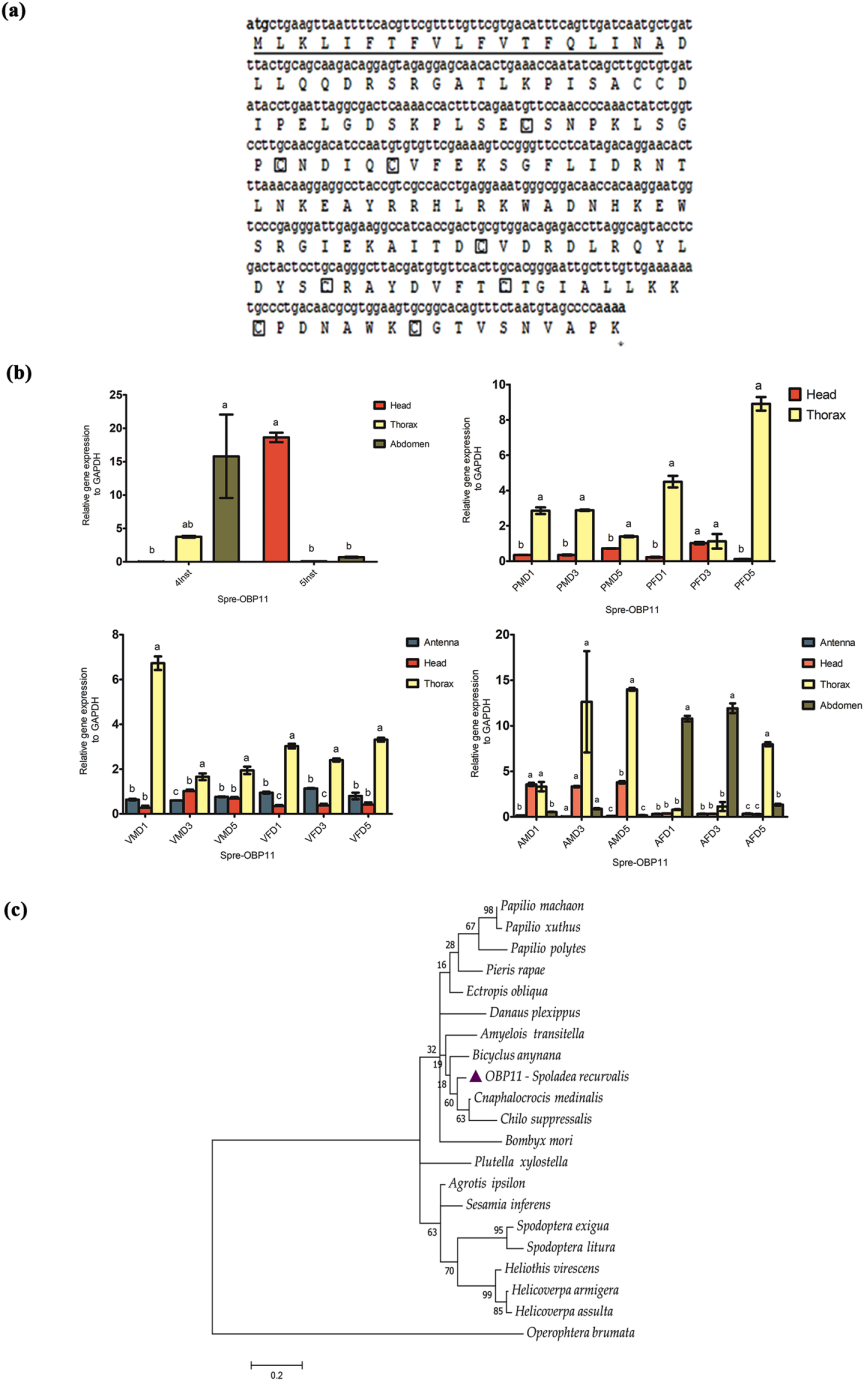
Schematic illustration of basic molecular characterization of odorant binding protein11 from *Spoladea recurvalis* (**a**) Nucleotide and deduced amino acid sequence of OBP11.The conserved cysteine residues are marked in square box. Predicted signal peptide was highlighted in underline. (**b**) qRT-PCR analysis shown the expression of Spre-OBP11 relative to each development stages (Inst-Instar; PMD-Pupae male day; PFD-Pupae female day; VMD-virgin male day; VFD-virgin female day; AMD-Mated adult male day; AFD-Mated adult female day). GAPDH was used as an internal control. Bar represents ±standard error. Different letters (**a**,**b**,**c**) shows the significant differences (P < 0.05). (**c**) Maximum Likelihood tree was constructed based on odorant–binding protein 11 the amino acid sequence using MEGA 6.0 with default settings model JTT + T inferred from 1000 replicates.

Based on the number and conserved cysteine location, *S. recurvalis* OBP11 and GOBP1 belong to sub family “plus-C” class, whereas partial sequence of PBP is a typical odorant binding protein and OBP4 (JHP) belongs to Takeout protein (Table 2). The open reading frame (ORF) of OBP11 was 471 bp, which encoded 157 amino acid residues, with predicted signal peptide cleavage site between 19 and residue 20 (Fig. 4a). Sequence alignment of Spre-OBP11 with corresponding proteins from other insects was listed in Supplementary Table S1. Among them, Spre-OBP11showed highest expression primarily in thorax and abdomen of 4^th^ instar larvae and higher in head of 5^th^ instar larva, whereas moderately lower in thorax and abdomen of 5^th^ instar larvae (Fig. 4b). Abundant gene expression was also found in thorax of male and female pupae of all ages, whereas its expression was moderate in male and female pupae head tissues. Furthermore, similar expression patterns were noticeably observed in unmated adult thorax tissue of all ages and moderately lower expression of Spre-OBP11 was observed in unmated male and female adult of antennal tissue. Spre-OBP11 was expressed higher in head and thorax of mated male on day 3 and 5, in the female abdomen on day 1 and 3, and female thorax on day 5 (Fig. 4b). However, there was no obvious Spre-OBP11 gene expression noticeable in antenna of mated male and female of all ages. Spre-OBP11 showed high amino acid identity with two Crambidae moths, *Cnaphalocrocis medinalis* (85%) and *Chilo suppressalis* (76%), followed by the Nymphalidae butterfly, *Danaus plexippus* (76%). Phylogenetic analysis showed that *S. recurvalis* had a high homology to *C. medinalis* belonging to the Plus-C OBP sub-family (Fig. 4c).

**Table 2:** Amino acid sequence characterization of putative PBP/OBPs identified from *S. recurvalis*.

*Spoladea recurvalis*	Subfamily	Signal Peptide	Sequence length	Protein core region
OBP11	Plus-C	Yes	471	C1-X_8_-C2_-_X_4_-C3-X_43_-C_4_-X_12_-C5-X_7_-C6-X_8_-C7-X_6_-C8
GOBP1	Plus-C	Yes	498	C1-X_30_-C2_-_X_3_-C3-X_42_-C_4_-X_10_-C5-X_8_-C6-X_4_-C7
PBP^p^	Classic OBP	—	177	C1-X_30_-C2-X_3_ -C3-x_7_
OBP4 (JHP)	TOP	Yes	762	C1-X-C2

*‘X’- denotes any amino acid; superscript p refers partial sequence*.

**Figure 5:**
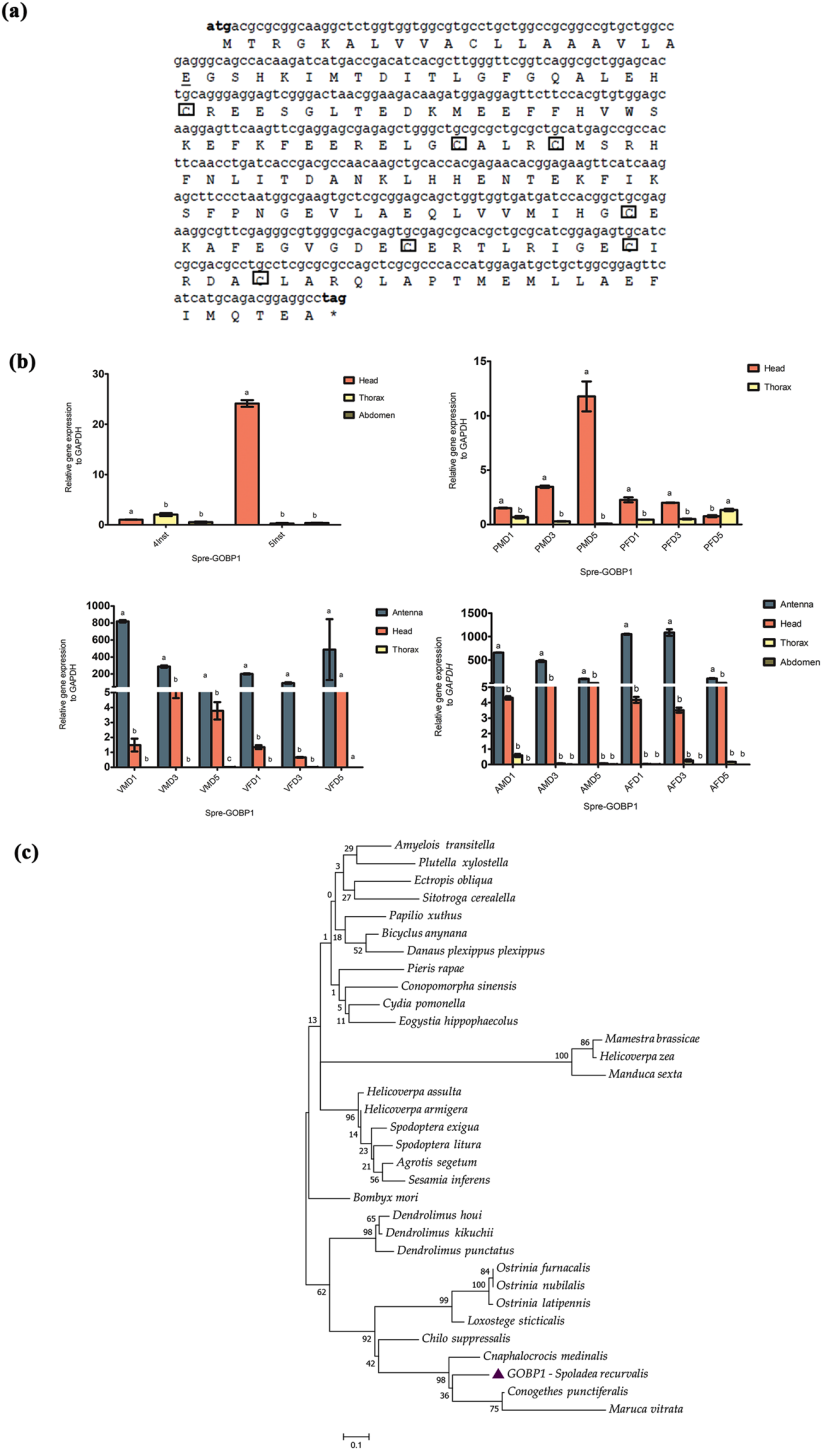
Schematic illustration of basic molecular characterization of general odorant binding protein1 (GOBP1) from *Spoladea recurvalis* (**a**) cDNA and derived amino acid sequence of Spre-GOBP1.The conserved cysteine residues are marked in square box. Predicted signal peptide was highlighted in underline. (**b**) qRT-PCR analysis shown the expression of Spre-GOBP1 relative to each development stages.(Inst-Instar; PMD-Pupae male day; PFD-Pupae female day; VMD-virgin male day; VFD-virgin female day; AMD-Mated adult male day; AFD-Mated adult female day).GAPDH was used as an internal control. Bar represents ±standard error. Different letters (**a**,**b**,**c**) shows the significant differences (P < 0.05). (**c**) Maximum Likelihood tree was constructed based on general odorant–binding protein 1 the amino acid sequence using MEGA 6.0 with default settings model LG + G inferred from 1000 replicates.

**Figure 6:**
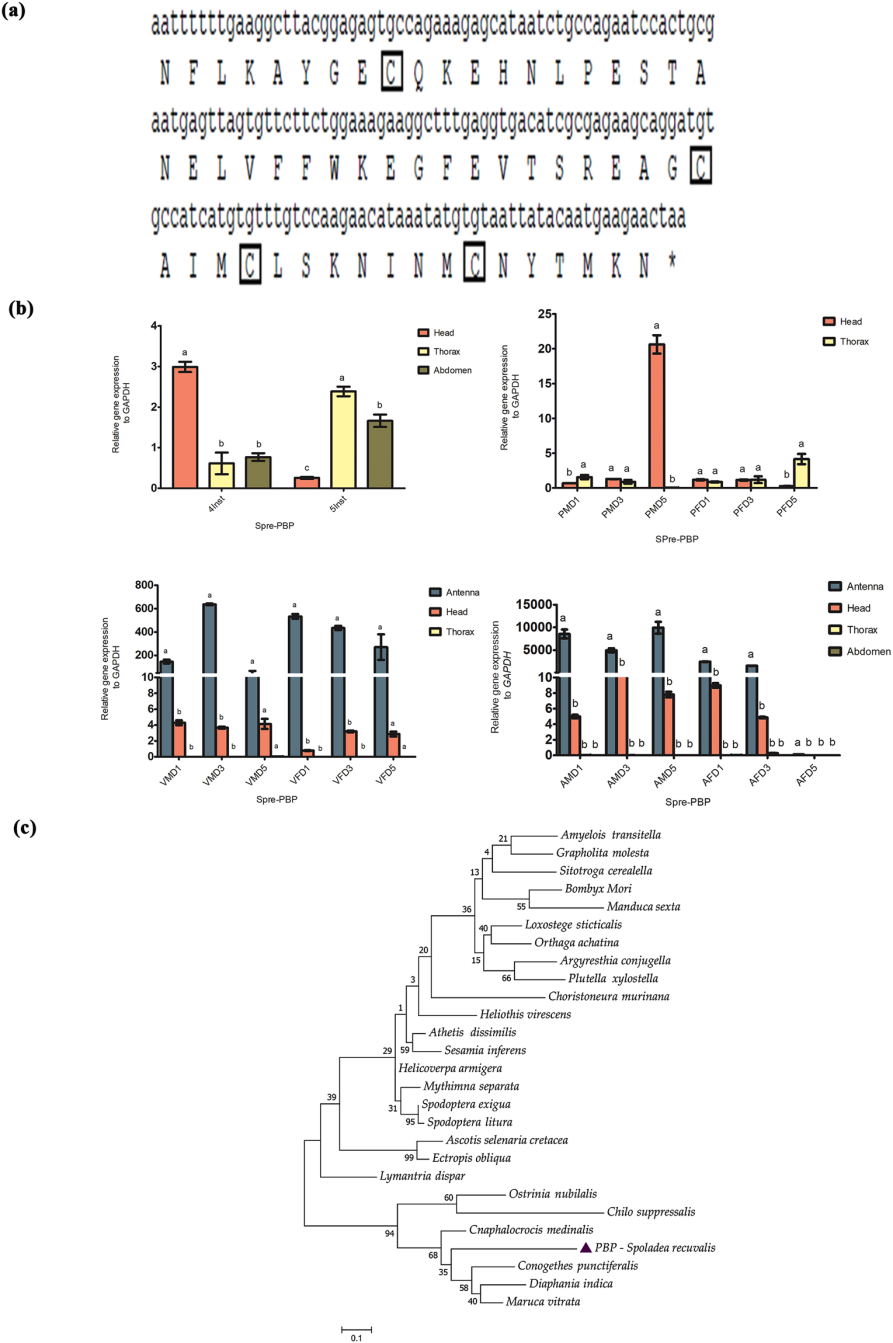
Schematic illustration of basic molecular characterization of pheromone binding protein (PBP) from *Spoladea recurvalis*. (**a**) 5′ missing partial cDNA and derived amino acid sequence of Spre-PBP. The conserved cysteine residues are marked in square box. (**b**) qRT-PCR analysis shown the expression of Spre-PBP relative to each development stages.(Inst-Instar; PMD-Pupae male day; PFD-Pupae female day; VMD-virgin male day; VFD-virgin female day; AMD-Mated adult male day; AFD-Mated adult female day). GAPDH was used as an internal control. Bar represents ±standard error. Different letters (**a**,**b**,**c**) shows the significant differences (P < 0.05). (**c**) Maximum Likelihood tree was constructed based on Pheromone binding protein the amino acid sequence using MEGA 6.0 with default settings model LG + G + I inferred from 1000 replicates.

The full-length sequence of GOBP1 was 498 bp, encoded by 166 amino acids. Predicted signal peptide was found at the N-terminal position between 19 and 20 (Fig. 5a). The deduced amino acids were aligned, revealing conserved cysteine residues, and it belonged to class “Plus-C” sub family (Fig. 5a). We observed that Spre-GOBP1 showed significantly higher expression particularly in the head of 5^th^ instar larvae and pupae of all ages (Fig. 5b). We observed significant Spre-GOBP1 gene expression in unmated and mated adult antennal and head tissues of male and female of all ages (Fig. 5b). Spre-GOBP1 has high amino acid sequence identity with three other Crambidae moths, *C. medinalis* (82%), *Conogethes punctiferalis* (77%) and *M. vitrata* (70%) (Supplementary Table S2). As indicated by the phylogenetic tree based on amino acid identity percentage, *C. medinalis*, *C. punctiferalis* and *M. vitrata* clustered together forming a single clade (Fig. 5c). The partial sequence (177 bp) of Spre-PBP was aligned and the sequence identity typically characterizes an odorant binding protein (Fig. 6a). Amino acid sequence alignment revealed high sequence similarity conserved among three other Crambidae moths, *C. medinalis* (70%), *Diaphania indica* (66%), and *M. vitrata* (64%) (Supplementary Table S3). Spre-PBP gene expression showed that the expression was found to be higher in head of 4^th^ instar larvae, and the level of expression was further observed to be higher in thoracic and abdomen region of 5^th^ instar larvae (Fig. 6b). Higher expression of Spre-PBP gene was significantly found in the head of male pupae on day 5 (Fig. 6b). Furthermore, Spre-PBP gene expression was significantly observed in antennal and head tissues of unmated and mated adults of all ages (Fig. 6b). Phylogenetic analysis was shown in Fig. 6c. Spre-PBP was compared with PBPs of other insect species and the results indicated that significantly high degree of conservation among lepidopterans evolved possibly from a common ancestor.

**Figure 7:**
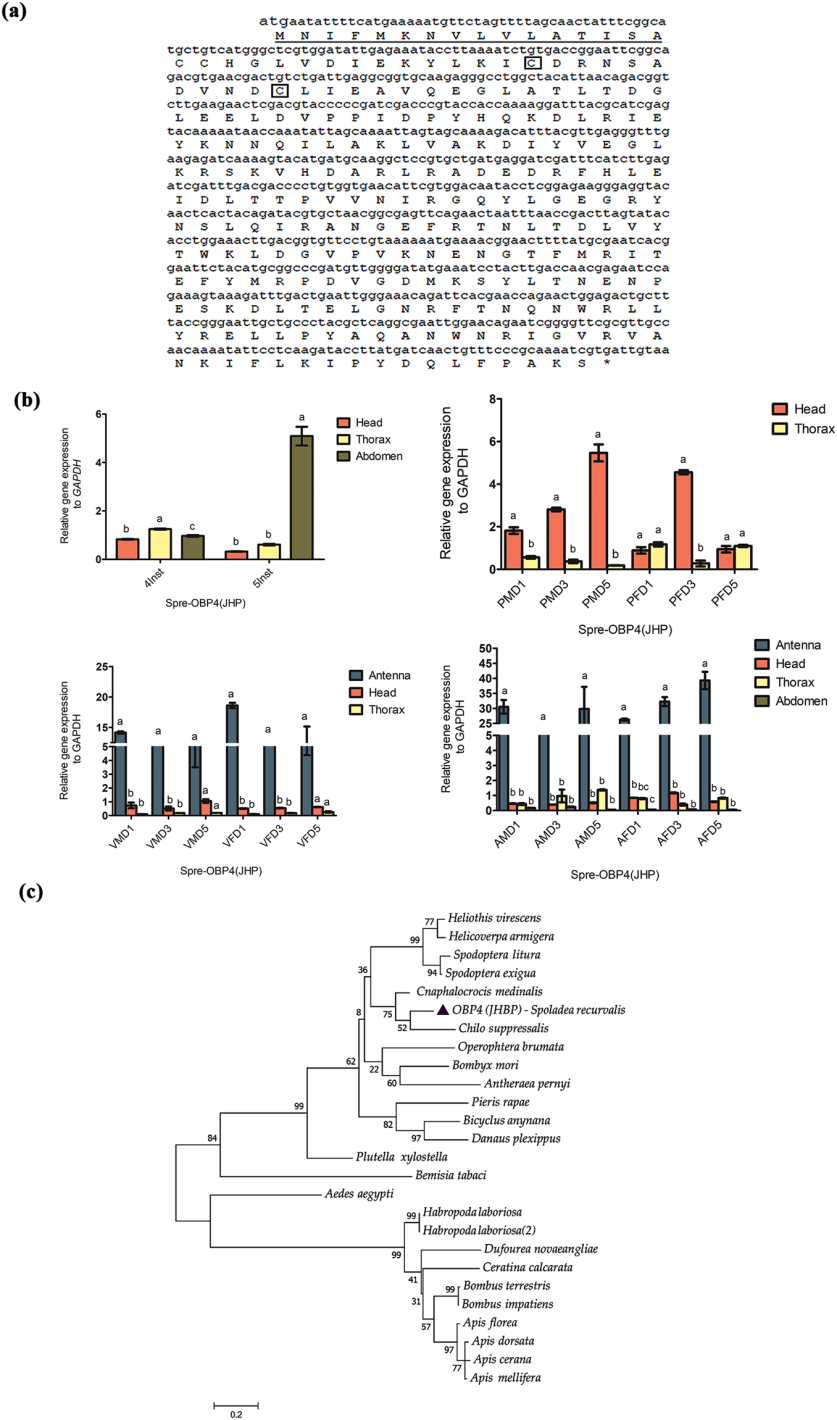
Schematic illustration of basic molecular characterization of odorant binding protein4 (OBP4) from *Spoladea recurvalis*. (**a**) cDNA and derived amino acid sequence of Spre-OBP4.The conserved cysteine residues are marked in square box. Predicted signal peptide was highlighted in underline. (**b**) qRT-PCR analysis shown the expression of Spre-GOBP1 relative to each development stages (Inst-Instar; PMD-Pupae male day; PFD-Pupae female day; VMD-virgin male day; VFD-virgin female day; AMD-Mated adult male day; AFD-Mated adult female day). GAPDH was used as an internal control. Bar represents ±standard error. Different letters (**a**,**b**,**c**) shows the significant differences (P < 0.05). (**c**) Maximum Likelihood tree was constructed based on odorant–binding protein 4 the amino acid sequence using MEGA 6.0 with default settings model LG + G inferred from 1000 replicates.

Similarly, the full-length open reading frame of Spre-OBP4 was 762 bp, encoding 253 amino acids. Predicted signal peptide and cleavage site position lies on 20 and 21 (Fig. 7a). Multiple sequence alignment and phylogenetic analysis showed that Spre-OBP4 has close homologues with two Crambidae moths, *C. medinalis* (86%), and *C. suppressalis* (79%), and a Pyralidae moth, *Amyelois transitella* (69%) (Supplementary Table S4). Spre-OBP4 sequence was similar to a juvenile hormone protein, which is a member of the odorant binding protein. Spre-OBP4 was primarily expressed in abdomen (Fig. 7b). The transcript levels were expressed in higher amounts in the head of pupae. More specifically, Spre-OBP4 was expressed in higher amounts in antenna of both unmated and mated adults of all ages (Fig. 7b). The resulting phylogenetic tree showed that Spre-OBP4 sequence clustered closely with the sequence of *C. medinalis* and *C. suppressalis* (Fig. 7c).

## Discussion

The production and release of sex pheromone in butterflies and moth species were mostly regulated by the neuropeptide, pheromone biosynthesis activating neuropeptide (PBAN) that plays various biological processes, potentially adapted to control insect pests^4,37^. For the first time, we are reporting here the characterization of Spre-DH-PBAN gene, binding proteins such as PBP and OBPs, and their differential and tissue-specific expression patterns. The Spre-PBAN flanked by three essential amino acids at its N terminus (G-K, G-R, G-R-R) and three other basic amino acids at its C terminus (G-R, G-R-K or G-R), which were believed to be the site for prohormone processing. The endoproteolytic cleavage, and glycine usually provide the amino group for amidation (Fig. 1)^38–40^, as reported for other DH-PBAN or PBAN that shared conserved pentapeptide sequence FXPR/KL at the C-termini^16–18^. Thus, amidation and physiological activity of DH and PBAN were essential, mainly for the members of Lepidoptera, Hymenoptera, Diptera, and Coleoptera^41^. Notably, the cDNA encoding all five neuropeptides (Spre-DH homologue, Spre-α-NP, Spre-β-NP, Spre-PBAN, and Spre-γ-NP) in this study were identical to the members of Lepidoptera^17,18,27^. The presence of 6 exons interrupted by five introns of DH-PBAN has been reported for other genera, including Marvi-DH-PBAN, since these gene products were the precursor of sex pheromone biosynthesis activating neuropeptide^16,18^ (Fig. 1).

The DH neuropeptide that we observed contains a conserved C-terminal motif, WFGPRL, which has been reported to play the role of inducing embryonic diapause in *B. mori*, and impeding pupal diapause in moths of the Noctuidae^42^. It is more obvious that Crambidae including *S. recurvalis* and *M. vitrat*a lacks histidine residues in the DH neuropeptide, which were common in Bombycoidea and Noctuoidea^18^ (Fig. 1). As in other lepidopterans, *S. recurvalis* was also found to have α-SGNP, which presents pheromonotropic activity, and can have interaction with the PBAN receptor, giving it similar functions as PBAN including diapause termination^10^. In addition, α-SGNP has the function for hindgut muscle stimulation and contraction in Madeira cockroach, *Leucophaea maderae*. Thus, α-SGNP was found to be present in insect orders besides Lepidoptera, contributing for other physiological functions, as well. Although α-SGNP was missing in insect orders Diptera, Hymenoptera, and Coleoptera, it was purported that α-SGNP domain can physically interact with β-SGNP to form one neuropeptide in fire ants, *Solenopsis invicta*^10,43^, but more evidences were needed to support this claim. Studies have shown that *M. vitrata*’s β-SGNP was different among the five peptides of the PBAN gene^18^, but not the most divergent as reported in *M. sexta* and *P. xylostella*^19,44^. Conversely in our study, *S. recurvalis* showed that β-SGNP has high similarity with *M. sexta*. Previous studies reported that the sequence of γ-SGNP has the higher resemblance with the likes of Noctuoidea, Pyraloidea, and Bombycoidea^17,18^. However, a variant C-terminal pentapeptide sequence LTPRLamide was identified in the study, and it was found to exist in *Clostera anastomosis* and *P. xylostella*. It has been shown that PBAN protein contains a highly conserved YFSPRL motif among the lepidopteran species constituting signature sequence for PK/PBAN family^6,7^. However, a variant C-terminal PBAN sequence showed asparagine substituted with serine in *M. vitrata*, revealing that this substitution was not commonly found in Pyraloidea super family (including this current study) (Fig. 1), which was associated with the pheromonotropic activity^16,18^ (Fig. 1). The complete coding and non-coding sequences of Spre-DH-PBAN gene from this study revealed that approximately 4.2 kb contains six exons and five introns with conserved intron position within the codon regions (Fig. 2). Similar reports of DH-PBAN genomes from *B. mori*^45^, *M. vitrata*^18^, and *O. nubilalis*^16^ had also been published, revealing that different pattern of exon and intron length and GC content could determine the evolutionary forces influenced by natural selection^46^.

The biosynthesis and release of DH-PBAN neuropeptides mostly arise from the sub-esophageal ganglion (SG) of moth species^4^. In our research, DH-PBAN was notably expressed in higher amounts in the abdominal region of 5^th^ instar (5INS) larvae suggesting its essential role in preparation for pupation^20^ (Fig. 3b). Previously, PBAN was widely recognized to accelerate pupation and cuticular tanning for color polymorphism^7,11^. Furthermore, PBAN has been shown to be associated with migrating ganglion cells in Lymantria dispar during development of larvae in an anteroposterior direction towards the abdomen^47^. Therefore, we hypothesized that Spre-PBAN was likely to function similarly during its development from larva to pupa stage. The Spre-PBAN gene expression during mid- and late-pupal stages implied physiological function of their role in stimulating the preparation for adult development, which was congruent with previous findings^18^, but contrary to *M. sexta* and *A. pernyi*^19,48^. In both virgin male and female moths, PBAN gene expressions were higher in head indicating the commencement of PBAN synthesis in sub-esophageal ganglion, followed by sex pheromone synthesis that attracts males for mating^5,18^ (Fig. 3b). After mating, females maintain PBAN transcripts in their head and thoracic region at a high-level due to the necessity of keeping the sex pheromone biosynthesis for multiple mating, circadian regulation as it is that FXPRL peptides in conjunction with the stimulation of pheromone can engage in other bioactivities^11,49^. However, evidences also suggested that mating may also inhibit pheromone production in females, which may be associated with cessation of PBAN release by hormonal and neural mechanism^49^. In *M. vitrata* and *C. transversa*, it was previously found that expression levels of PBAN in female were notably lower than those in male adults^17,18^. This low PBAN difference in females was thought to spring from the minimal need for the PBAN among the female pod borers. However, this is not observed in our current findings (Fig. 3b), and hence such variations could be species-specific.

In the present study, we have chosen four putative PBP/OBPs to understand the functional differentiation of PBPs and/or OBPs in *S. recurvalis*. Moreover, our study chose these PBP/OBPs given the fact that different studies had varying opinions on insect peripheral olfactory reception^31^, and we wanted to bridge the knowledge gap using molecular characterization. In our study, Spre-OBP11 showed differing expressions in the various developmental stages of the insect. In fact, these observations were not noted in the other studies (Fig. 4b). During larval stages, we presumed that Spre-OBP11 might assist in host plant discrimination and sense nutrient sources. The differences in Spre-OBP11 expression in antenna, head and thorax of virgin female and male tissues as well as post-mating head and thorax tissues may have plausibly been related to the perception of pheromone in addition to host plant volatile compounds. In post mating, female abdomen tissues were shown to abundantly express Spre-OBP11 compared to male tissues. Study from orthologue rice leaf-folder (*C. medinalis*) showed similar results that OBP was abundantly expressed in female tissues, in which CmOBP was used to find suitable host leaf surfaces prior to female oviposition^50,51^. It is conceivable that Spre-OBP11 may have a similar function in ovipositing *S. recurvalis* female moths. Hence, it is worth investigating such a behavior response in *S. recurvalis* to OBPs in future research.

In this study, GOBP1 expressed abundantly in antenna and head of both virgin and mated female moths, which implies that Spre-GOBP1 likely involved in chemoreception to find suitable interspecific signals such as sex pheromone components and host plant volatiles (Fig. 5b). In *M. vitrata*, GOBP1 and GOBP2 were reported to be involved in chemoreception considering its broader role in detecting volatile odor molecules from host plants^52^. Another study revealed that two GOBPs of *Spodoptera exigua* were sex-equivalent supposing that Spre-GOBPs were likely to have similar role in host volatile recognition^53^. Our qRT-PCR results indicated that PBP was upregulated in head of 4^th^ instar larvae and male pupae. Spre-PBP expressed in the antenna and heads of virgin moths of both sexes. However, Spre-PBP was highly expressed in mated males than in the females (Fig. 6b). We hypothesized that Spre-PBP involved in auto-detection of sex pheromone compounds by female moths in order to ensure multiple mating, which has been discussed in previous studies^32^. Some studies have suggested that dendrites that adjoining to the soluble PBPs/OBP in sensillum lymph were anticipated to relocate the dendrite membrane of the sensory neurons from the hydrophobic pheromone^22^. Other studies reported that PBPs attributed to certain receptions of sex-pheromone components, although PBPs were capable of binding to numerous pheromone components^22,25,53–55^. This was because of the fact that the complex peripheral mechanism in the olfactory system possesses the ability to purify the selection. Hence, the PBP/OBP family might have the additional function acting as transporters in the non-chemosensory tissues^56^. Thus, Spre-PBP may also have extensive functions besides sensing the pheromone compounds, which are yet to be studied. Similarly, results from Spre-OBP4 showed that the expression was abundant in tissues of 5^th^ instar larval abdomen and pupal head stages of both sexes (Fig. 7b). We observed a higher expression pattern between pre-mated and post-mated adult tissues, especially mated male and female antennal tissues showing higher expression, whereas head and thorax tissue had moderately lower level of expression in all ages (Fig. 7b). It is interesting to note that Spre-OBP4 is a member of Takeout protein. It is unknown whether Spre-OBP4 has a binding property to small lipophilic juvenile hormone molecule, which might be regulated by starvation and timing or duration of host volatile odor signal^57^. However studies from *Phormia regina* and *B. mori* suggest that this odorant binding protein – juvenile hormone protein (OBP4-JHP) belongs to Take Out Protein subfamily particularly localized around axillary cell membranes and thought to be involved in early perception of the chemical signals in both gustatory and olfactory system^58,59^.

Overall, our results of selected PBP/GOBP/OBP prompted us to speculate that *S. recurvalis* exhibit some common biological functions when compared to other lepidopteran species to locate the host, recognition of odorous compounds including plant volatiles and pheromones, and circadian rhythm prior to oviposition. Considering that the selected *S. recurvalis* PBP and GOBP/OBP were close homologs to rice leaf-folder (*C. medinalis*), highly conserved among insect orders, presumably derived from single gene duplication event initiated from a common ancestor under environmental pressure across 250 million years^60,61^. Possibly, differential expression among the four putative binding protein genes can have developed some unique characteristics in chemosensory system of *S. recurvalis* to become adapted to different ecosystem in which host plants were present. Hence, characterization of these genes may be useful to investigate the population diversity in *S. recurvalis*, as well as to target the control of this pest based on genetic manipulation of olfactory system.

## Methods

### Insect Rearing and Tissue Collection

*Spoladea recurvalis* moths were collected from amaranth crops in Shanhua, Tainan, Taiwan. The insects were mass-reared following the method described previously^34^, and tissue samples were collected as described in Chang *et al*.^18^. Whole insects in different life stages were collected as fourth-and-fifth instar larvae, pupae and virgin as well as mated male and female adults. Antenna, head without antenna, thorax, and abdomen from male and female moths were excised and stored in RNA later.

### RNA Extraction and 5′ And 3′ Rapid Amplification of cDNA Ends (5′ and 3′ RACE)

Owing to our great interest to understand the biological functions of PBAN and PBP/OBPs in *S. recurvalis* and molecular basis of pheromone olfactory system, all sequence information corresponding to PBAN and selected PBP/OBP transcripts were obtained from the transcriptome library^62^ (Supplementary Table. S5). We included different life-stages (5^th^ instar larvae, pupae, virgin and mated adults) for studying the gene expression of PBAN and PBP/OBPs. The sum of RNA was separated from the other tissues dissected from different life-stages described above using the “Total RNA mini kit” (Qiagen Ltd,) from Venlo-Netherland according to the manufacturer’s protocol, with on-column DNase I treatment. A nano-drop spectrophotometer (Thermo Scientific) at 260 nm was used to quantify RNA by absorbance measurement. First, sequences showing homology with OBP genes were used to design gene-specific and nested reverse and forward primers to obtain 5′ and 3′ ends of the full-length cDNAs by rapid amplification of cDNA ends (RACE method). The sequences and primers used for the 5′ and 3′ RACE are listed in Supplementary Table S6. All the steps of RACE were performed using 5′RACE RT-PCR system (Invitrogen) instructions. Total RNA from the fifth instar larval head was used as a template for generating 5′ and 3′ RACE ready cDNAs. Following ligation of the 5′ RACE adapter oligomer, reverse transcription with Superscript III was performed using oligonucleotide as primer. Besides, a unique gene-specific DNA fragment was increased in each case. After amplification, the 5′RACE and 3′RACE PCR products were separated in a 2.5% agarose gel with ethidium bromide (EtBr) staining. The gel Slices containing DNA fragments were purified using an agarose gel DNA purification kit (Geneaid, Taiwan), according to the manufacturer’s instructions, and were subjected to sequencing.

### Quantitative real-time PCR (qRT-PCR) analysis

Quantitative RT-PCR was carried out with a Bio-Rad CFX system in the final reaction volume of 10 μLcontaining 2.5 μL cDNA of nuclease-free water, 5x SYBER Green Real-time PCR Master Mix and 10 μM each of forward and reverse primers (Supplementary Table. S6). The thermal cycling conditions were noted as follows: Initial denaturation 95 °C for 3 mins, 95 °C for 10 sec, Annealing 60 °C for 45 sec, following 40 cycles qRT-PCR was performed for three biological replicates. GAPDH was used to normalize the gene expression. The relative expression levels of the genes were calculated using the 2^ΔΔCT^ method^63^, which determines the difference in the CT between the control GAPDH product and the target gene product. Relative fold change for each gene was compared to respective stage of tissue and  day for normalization.

### Molecular and Phylogenetic Analyses of Sequence Data

Nucleic acid and deduced amino acid sequences were determined by ORF finder (http://www.ncbi.nlm.nih.gov/gorf/gorf.html). To identify the putative orthologs of *S. recurvalis*, similarity sequence information was retrieved using BLASTn and BLASTp. The ClustalW^64^ module in MEGA6^65^ software was used to align multiple sequences. The cDNA encoding PBAN possess five neuropeptides: 24-amino acid residue of DH motif (V22 to L45), and the other four neuropeptides such as 36 aa PBAN (G175 to R166), three short SGNPs, α-7-aa (K95 to G102), β-20 aa (R105 to G126), and γ-8 aa (R166 to G175), were individually aligned using Clustal W, and compared against NCBI database to identify homologs of other insects. The raw output of the multiple sequence alignment was refined to minimize insertion/deletion events. The evolutionary relationships were inferred using the Maximum-likelihood method for interspecific and intraspecific species using MEGA6 with the best-fit phylogenetic model^18^. Branch supports were surveyed by bootstrapping 1000 times. Putative signal peptide was predicted using SIGNAP 4.1 program with default settings^66^.

### Statistical Analysis

The statistical analysis was done using the Graph Pad Prism 5.0 (Graph Pad Software La Jolla, CA, USA). Results of qRT-PCR were analysed using 2^−ΔΔCt^ method^63^ to calculate the relative gene expression of three biological and three technical replicates (n = 6) for each target gene. Values were given as mean ± standard error (SE). Significant differences were indicated by different letters (a, b, c, P < 0.05). Analysis of variance (ANOVA) was conducted to evaluate significant differences in expression level for each gene among different tissues and developmental stages of males and females, followed by Tukey’s ‘Honest Significance Difference (HSD) test with the critical level of α = 0.05.

## Supplementary information

Amino acid sequence similarities of binding proteins and list of transcripts as well as primers

## Data Availability

All data generated or analysed during this study are included in the published article (and its Supplementary information files).
