# Supplementary material for: Sex-specific spatial and temporal gene expressions of Pheromone biosynthesis activating neuropeptide (PBAN) and binding proteins (PBP/OBP) in Spoladea recurvalis
Source: Sci Rep. 2019 Mar 5;9:3515. doi: 10.1038/s41598-019-39822-x (PMC6401106; doi:10.1038/s41598-019-39822-x)
Supplement: Supplementary file 1 — Amino acid sequence similarities of binding proteins and list of transcripts as well as primers [file 41598_2019_39822_MOESM1_ESM.pdf]

Sex-specific spatial and temporal gene expressions of Pheromone biosynthesis activating neuropeptide (PBAN) and binding proteins (PBP/OBP) in *Spoladea recurvalis* (Lepidoptera: Crambidae)

Rajendran Senthilkumar[1], and Ramasamy Srinivasan[2,*]

[1, 2] World Vegetable Center, Shanhua, Tainan 74151, Taiwan

Supplementary information

Supplementary Table S1 Amino acid sequence similarities of odorant binding protein11 (OBP11) from *Spoladea recurvalis* and other lepidopteran insect species

Supplementary Table S2 Amino acid sequence similarities of general odorant binding protein 1 (GOBP1) from *Spoladea recurvalis* and other lepidopteran insect species

Supplementary Table S3 Amino acid sequence similarities of pheromone binding protein (PBP) from *Spoladea recurvalis* and other lepidopteran insect species

Supplementary Table S4 Amino acid sequence similarities of odorant binding protein juvenile hormone protein (OBPJHP) from *Spoladea recurvalis* and other lepidopteran insect species

Supplementary Table S5 Selective list of PBP/OBPs transcripts identified from transcriptome library of *Spoladea recurvalis*

Supplementary Table S6 List of primers used in this study

Supplementary Table S1 Amino acid sequence similarities of odorant binding protein11 (OBP11) from *Spoladea recurvalis* and other lepidopteran insect species

Species	Homology Identity	Accession number
Spoladea recurvalis	-	-
Cnaphalocrocis medinalis	85%	ALT31641.1
Sesamia inferens	81%	AGS36744.1
Bicyclus anynana	80%	XP_023941241.1
Dendrolimus punctatus	77%	ARO70191.1
Danaus plexippus plexippus	76%	OWR44654.1
Chilo suppressalis	76%	AGK24580.1
Pieris rapae	73%	XP_022114205.1
Plutella xylostella	73%	ANC60176.1
Ectropis obliqua	71%	ANA75017.1
Agrotis ipsilon	70%	AGR39569.1
Bombyx mori	70%	XP_004932705.1
Spodoptera exigua	70%	AKT26495.1
Amyelois transitella	69%	XP_013191861.1
Spodoptera litura	69%	XP_022828050.1
Papilio polytes	68%	XP_013134166.1
Papilio machaon	67%	KPJ17152.1
Papilio xuthus	67%	KPI94076.1
Heliothis virescens	66%	PCG63721.1
Helicoverpa armigera	65%	XP_021186226.1
Helicoverpa assulta	65%	ASA40073.1
Operophtera brumata	51%	KOB70533.1
Dendrolimus kikuchii	29%	AII01008.1

Supplementary Table S2 Amino acid sequence similarities of general odorant binding protein 1 (GOBP1) from *Spoladea recurvalis* and other lepidopteran insect species

Species	Homology Identity	Accession number
Spoladea recurvalis	-	-
Cnaphalocrocis medinalis	82%	AFG72996.1
Conogethes punctiferalis	77%	APG32536.1
Maruca vitrata	70%	ALM04194.1
Loxostege sticticalis	68%	ACB47481.1
Chilo suppressalis	68%	ACJ07126.1
Ostrinia furnacalis	68%	BAV56786.1
Ostrinia nubilalis	68%	BBB15959.1
Ostrinia latipennis	67%	BBB15960.1
Amyelois transitella	64%	ACX47893.1
Bombyx mori	63%	NP_001037496.1
Dendrolimus houi	63%	AGJ83358.1
Agrotis segetum	63%	ABI24159.1
Cydia pomonella	63%	AFP66957.1
Dendrolimus punctatus	63%	ARO70211.1
Dendrolimus kikuchii	63%	AGJ83357.1
Helicoverpa assulta	62%	AAW65076.1
Sesamia inferens	62%	AGS36742.1
Pieris rapae	61%	XP_022118428.1
Eogystia hippophaecolus	61%	AOG12859.1
Conopomorpha sinensis	61%	AHY86493.1
Ectropis obliqua	60%	ACN29680.1
Papilio xuthus	59%	KPJ00908.1
Bicyclus anynana	59%	XP_023936603.1
Sitotroga cerealella	59%	AII15787.1
Spodoptera exigua	58%	ACY78412.1
Helicoverpa armigera	57%	AAL09821.1
Plutella xylostella	56%	ABW05104.2
Spodoptera litura	45%	AKI87961.1
Mamestra brassicae	44%	AAC05703.2
Manduca sexta	42%	AAG50015.1

Supplementary Table S3 Amino acid sequence similarities of pheromone binding protein (PBP) from *Spoladea recurvalis* and other lepidopteran insect species

Species	Percentage homology	Accession number
Spoladea recurvalis	-	-
Cnaphalocrocis medinalis	69.811	AGI37364.1
Diaphania indica	66.038	BAG71419.1
Conogethes punctiferalis	64.151	ALC76550.1
Maruca vitrata	64.151	AGS46555.1
Conogethes punctiferalis	64.151	AKQ98177.1
Ostrinia nubilalis	60.377	ADT78492.1
Ostrinia latipennis	62.264	BBB15984.1
Helicoverpa assulta	45.614	AAW65077.1
Athetis dissimilis	46.429	ALJ93809.1
Helicoverpa armigera	43.86	CAC08212.1
Spodoptera exigua	45.283	ABK41049.1
Helicoverpa zea	43.86	AAC36315.1
Spodoptera exigua	45.283	AAS55551.2
Manduca sexta	49.057	AAF16715.1
Bombyx mori	50.943	NP_001037494.1
Plutella xylostella	43.396	BAG71422.1
Ascotis selenaria cretacea	45.283	BAF64703.1

Supplementary Table S4 Amino acid sequence similarities of odorant binding protein juvenile hormone protein (OBPJHP) from *Spoladea recurvalis* and other lepidopteran insect species

Species	Homology Identity	Accession number
Spoladea recurvalis	-	-
Cnaphalocrocis medinalis	86%	ALT31634.1
Chilo suppressalis	79%	ADD71058.1
Amyelois transitella	69%	XP_013191310.1
Eogystia hippophaecolus	68%	AOG12867.1
Heliothis virescens	68%	PCG78400.1
Helicoverpa armigera	67%	XP_021187373.1
Spodoptera litura	66%	XP_022821989.1
Spodoptera exigua	65%	AKT26500.1
Plutella xylostella	64%	XP_011554689.1
Bombyx mori	62%	BAH79159.1
Bicyclus anynana	61%	XP_023945715.1
Pieris rapae	61%	XP_022117429.1
Antheraea pernyi	61%	ARD05169.1
Papilio xuthus	59%	KPI94823.1
Danaus plexippus plexippus	57%	OWR51011.1
Operophtera brumata	56%	KOB78556.1

Supplementary Table S5 Selective list of PBP/OBPs transcripts identified from transcriptome library of *Spoladea recurvalis*

**Sequence id**	**Transcript**	**FKPM value**	**Length**	**Nr annotation**
comp85370_c0_seq1	GOBP1	5.06	600	*Cnaphalocrocis medinalis*
comp9036_co_seq1	OBP	321.3	592	*Danaus plexippus*
comp107388_c0_seq1	OBP	0.29	232	*Diaphania indica*
comp92600_co_seq1	OBP	0.83	509	*Danaus plexippus*

Supplementary Table S6 List of primers used in this study

Primer name	Primer pair (5' to 3')	
	**Forward**	**Reverse**
RT -PCR		
PBAN	5'ACATCTTATGGGAAGAAGAAACTTG-3'	5'TCGCTGTTTGTTACATTTAAAGT-3'
	5'-GTCTTACCTCGGCTGTTGATG-3'	5'-GGGAGTCGTAGAAGGGTAACTGA
	5'-TCAGTTACCCTTCTACGACTCC-3'	5'-CTACGTCATCATCAGAAGGTGTG-3'
	5'-CGGCACCGTTTACCAAGATA-3'	5'-CTTCGTACTAGCCGAATCTTCTC-3'
QPCR		
qGAPDH	5'CTGGAGTATTCACCACCATTGA-3'	5'-GACACCGCAGACGTACATAG-3'
qPCR PBAN	5'-CGGCACCGTTTACCAAGATA-3'	5'-GTACACTACGTCATCATCAGAAGG-3'
qPCR OBP11)	5'-AACTGTCTGGTCCTTGTAACG-3'	5'-CGGTGATGGCCTTCTCAAT-3'
qPCR (GOBP1)	5'-GGGACTAACGGAAGACAAGATG-3'	5'-TGATGAACTTCTCCGTGTTCTC-3'
qPCR (OBP4-JHP)	5'-CGTGCTGATGAGGATCGATTT-3'	5'-CCGTCAAGTTTCCAGGTGTAT-3'
qPCR PBP	5'-CCACTGCGAATGAGTTAGTGTTC-3'	5'-CTCCGTGCTGTTTCACAAAGTC-3'
RACE PCR		
OBP11	GGAGTAGAGGAGCAACACTGAAAC	GGAGTAGTCGAGGTACTGCCTAAG
Nested OBP11	CAAGACAGGAGTAGAGGAGCAAC	GAGGTACTGCCTAAGGTCTCTGT
PBP	TTTGAAGGCTTACGGAGAGTGC	GTTTCACAAAGTCGTTGGTCTTCCC
Nested PBP	ACGGAGAGTGCCAGAAAGAGC	CCTTCAGGATCTATCAGGTCCATC
